# Development of somatic hybrids *Solanum* × *michoacanum* Bitter. (Rydb.) (+) *S. tuberosum* L. and autofused 4*xS.* × *michoacanum* plants as potential sources of late blight resistance for potato breeding

**DOI:** 10.1007/s00299-013-1422-5

**Published:** 2013-03-24

**Authors:** P. Smyda, H. Jakuczun, K. Dębski, J. Śliwka, R. Thieme, M. Nachtigall, I. Wasilewicz-Flis, E. Zimnoch-Guzowska

**Affiliations:** 1 Plant Breeding and Acclimatization Institute-National Research Institute (IHAR-PIB), Młochów Research Centre, Platanowa 19, 05831 Młochów, Poland; 2 Julius Kühn-Institute, Federal Research Centre for Cultivated Plants, Institute for Breeding Research on Agricultural Crops, Erwin-Baur-Str. 27, 06484 Quedlinburg, Germany

**Keywords:** Potato species, Protoplast electrofusion, *Phytophthora infestans* resistance, Somatic hybridization

## Abstract

****Key message**:**

***Phytophthora infestans*** **resistant somatic hybrids of** ***S.*** **×** ***michoacanum*** **(+)** ***S. tuberosum*** **and autofused 4** ***x*** ***S.*** **×** ***michoacanum*** **were obtained. Our material is promising to introgress resistance from** ***S.*** **×** ***michoacanum*** **into cultivated potato background.**

**Abstract:**

*Solanum* × *michoacanum* (Bitter.) Rydb. (*mch*) is a wild diploid (2*n* = 2*x* = 24) potato species derived from spontaneous cross of *S. bulbocastanum* and *S. pinnatisectum.* This hybrid is a 1 EBN (endosperm balance number) species and can cross effectively only with other 1 EBN species. Plants of *mch* are resistant to *Phytophthora infestans* (Mont) de Bary. To introgress late blight resistance genes from *mch* into *S. tuberosum* (*tbr*), genepool somatic hybridization between *mch* and susceptible diploid potato clones (2*n* = 2*x* = 24) or potato cultivar Rywal (2*n* = 4*x* = 48) was performed. In total 18,775 calli were obtained from postfusion products from which 1,482 formed shoots. The Simple Sequence Repeat (SSR), Cleaved Amplified Polymorphic Sequences (CAPS) and Random Amplified Polymorphic DNA (RAPD) analyses confirmed hybrid nature of 228 plants and 116 autofused 4*x* *mch*. After evaluation of morphological features, flowering, pollen stainability, tuberization and ploidy level, 118 somatic hybrids and 116 autofused 4*x* *mch* were tested for late blight resistance using the detached leaf assay. After two seasons of testing three somatic hybrids and 109 4*x* *mch* were resistant. Resistant forms have adequate pollen stainability for use in crossing programme and are a promising material useful for introgression resistance from *mch* into the cultivated potato background.

## Introduction

*Phytophthora infestans* (Mont.) de Bary is one of the most important pathogens of the cultivated potato *Solanum tuberosum* L. (*tbr*) worldwide. It is responsible for the late blight disease, which results in 10–15 % reduction of the global production of potato tubers (Kamoun and Smart 2005; Park et al. 2009). Identification of new sources of resistance to *P. infestans* and introgression of novel resistance genes from wild potato species into the tetraploid potato genepool is a way to achieve the progress in breeding of the cultivated potatoes resistant to late blight (Zimnoch-Guzowska et al. 2003; Guo et al. 2010). Due to differences in the ploidy level or in the Endosperm Balance Number (EBN) many wild potato species cannot be used as late blight resistance donors in crosses with *tbr* (Borgato et al. 2007; Tiwari et al. 2010). According to the EBN hypothesis, some species, including *Solanum* has a specific EBN value, called effective ploidy, determined from crosses with standard species. Successful crosses require a 2:1 maternal:paternal EBN ratio in the hybrid endosperm (Johnston and Hanneman 1980). Somatic hybridization is an alternative method to classical sexual hybridization (Oberwalder et al. 2000; Wielgat and Wasilewska 2001). It enables obtaining variable materials with higher level of resistance and preserved biodiversity in the nuclear and cytoplasmic genomes (Thach et al. 1993; Davey et al. 2005). Somatic hybridization has a potential for introgression of mono- and polygenic traits from wild *Solanum* species into *tbr* genepool (Gavrilenko et al. 2003). Resistant to *P. infestans* somatic hybrids has been obtained between *tbr* and *S. circaeifolium* (Mattheij et al. 1992), *S. pinnatisectum* (Thieme et al. 1997; Polzerová et al. 2011), *S. bulbocastanum* (Helgeson et al. 1998), *S. nigrum* (Horsman et al. 2001), *S. berthaultii* (Bidani et al. 2007), *S. tarnii* (Thieme et al. 2008) and *S. cardiophyllum* (Thieme et al. 2010). Somatic hybrids obtained using *S. bulbocastanum* (Helgeson et al. 1998), *S. nigrum* (Horsman et al. 2001), *S. tarnii* (Thieme et al. 2008) and *S. commersoni* (Carputo et al. 2000) were backcrossed sexually to potato cultivars and exploited in potato breeding programs.

*Solanum* × *michoacanum* (Bitter.) Rydb. (*mch*) is a wild, 1EBN diploid (2*n* = 2*x* = 24) nothospecies, a relative to potato, which occurs on the area of Morelia in Michoacán State of Mexico (Hawkes 1990). In natural habitat it grows in damp grassy fields and among rocks, elevated 2,000–2,100 m above the sea level. *Mch* is tuber-bearing species originating from a spontaneous cross of *S. bulbocastanum* and *S. pinnatisectum* (Hawkes 1990) and it is morphologically intermediate between them. *Mch* is known as the source of resistance to *P. infestans* of both leaflets and tubers (Jakuczun and Wasilewicz-Flis 2004a; Zoteyeva et al. 2004, 2012). Clones suitable for the potato chips production were found within *mch* (Jakuczun and Wasilewicz-Flis 2004b). *Mch* clones resistant to *P. infestans* were selected (Jakuczun and Wasilewicz-Flis 2004a; Smyda et al. 2011). The *P. infestans* resistance gene *Rpi*-*mch1* was mapped on potato chromosome VII (Śliwka et al. 2012).

The present paper reports production of somatic hybrids *mch* (+) *tbr* and autofused 4*x mch* plants via electrofusion, which can be potential sources of late blight resistance in the cultivated potato breeding.

## Materials and methods

### Plant material

The parental forms used in electrofusion were: two clones of *mch*: 99-12/8 *(mch/*8*)* and 99-12/39 (*mch*/39), three diploid potato clones: DG 81-68, DG 88-89 and dH Bard, and the cultivar (cv.) Rywal. Clones *mch*/8 and *mch*/39 were resistant to *P. infestans*, which was determined before in laboratory detached leaf test in 4 years of evaluation. They derived from the accession VIR5763 of *mch*, which was received from the N. I. Vavilov Research Institute of Plant Industry (VIR) potato collection, St. Petersburg, Russia. DG 81-68, DG 88-89, dH Bard and cv. Rywal were susceptible to late blight and were chosen from the collection of IHAR-PIB, Młochów. DG 81-68 and DG 88-89 were hybrids of *tbr*, *S. chacoense* and *S. yungasense*. In addition, DG 88-89 had *S. gourlayi* in origin. They were male fertile, producing functional 2*n* male gametes, and useful for protoplast fusion with other diploids (Przetakiewicz et al. 2007). Dihaploid dH Bard derived from cv. Bard expressed male sterility, however, it can function well as a seed parent. cv. Rywal was resistant to PVY (Szajko et al. 2008). Plants of parental forms were maintained in vitro and propagated by nodal subculture on hormone-free MS medium (Murashige and Skoog 1962), 2 % sucrose, 0.75 % agar and 0.115 % NH_4_NO_3_. Selected forms were virus and bacteria-free and originated from in vitro gene bank. In order to establish in vitro plants of selected parental forms, the presence of common viruses of potato, bacterial and PSTV infection was checked in plants grown in greenhouse conditions. Pathogen-free forms were established in vitro by cutting apical buds and decontaminated with sterilizing agents. Plant material was immersed in the 70 % ethanol for 30 s and then immersed in 2 % solution of sodium hypochlorite for 4 min. Then material was washed with sterile distilled water three times for 5 min each time.

### Protoplast isolation, fusion, and culture

Protoplast isolation and fusion were done in Julius Kühn-Institute (JKI), Gross Lüsewitz, Germany according to protocols described by Thieme et al. (2008). About 1 g of leaflets from 3 to 4 weeks old in vitro plants was used for isolation of mesophyll protoplasts according to Mőllers et al. (1992). The enzyme solution containing 0.2 % macerozyme (Serva, Heidelberg, Germany) and 0.8 % cellulase (Serva, Heidelberg, Germany) was applied for digestion of the cellular walls. The purified protoplasts of *mch* and *tbr* at a density of 1 × 10^6^ pp/ml were mixed in a ratio of 1:1. 200 μl of this mixture was placed in lamellar fusion chamber. Fusion was achieved by applying first 18–20 V of an AC-field of 800 kHz for aligning the protoplasts, followed by two DC pulses of 120 V amplitude and 15 μs duration with a break of 2 s and AC-field of 10–20 s. Fusion of protoplasts was carried out using the CFA 500 electrofusion equipment of Krüss Company, Hamburg. Afterwards, modified VKM-medium (Binding and Nehls 1977) was added to suspension of fused protoplasts. The cultures were kept in a culture chamber at 25 °C in the dark. The microcalluses forming in the suspension were transferred to Cul-medium (Haberlach et al. 1985) and exposed to 16 h/day illumination (fluorescent light intensity: 55.5 μmol/m^2^/s^1^) at 25 °C. After 4 weeks, calluses were transferred on the RJM regeneration medium (Masson et al. 1988). Only one shoot was excised from each callus and rooted on the MS medium. One in vitro copy was preserved for each plant, second copy was planted in pot and propagated in the greenhouse. Plants were covered by glass cap for first 2 weeks of growing in the greenhouse.

### DNA analysis

Genomic DNA was extracted from 200 mg fresh, young leaves of greenhouse-grown plants using the GeneElute™ Plant Genomic DNA Miniprep Kit (Sigma). The hybrid nature of somatic hybrids was confirmed by SSR, CAPS and RAPD markers.

#### SSR markers

The hybrid nature of plants from *mch*/39 (+) DG 81-68 fusion combination was proved by three SSR markers: ST13ST (localized on chromosome V by Sandbrink et al. 2000), STI 057 (localized on chromosome IX by Feingold et al. 2005) and STM 1049 (mapped to chromosome I, by Ghislain et al. 2004) based on Provan et al. (1996) protocol. PCR amplicons were separated on 6 % polyacrylamide denaturing gel in Sequin-Gen GT sequencing cell (Bio-Rad Laboratories, Inc.) and visualized by silver-staining method (Thieme et al. 2008).

#### CAPS and RAPD markers

The CAPS marker C2_At5g51970 (Tomato-EXPEN 2000, SGN 2012) and RAPD primer OPA11 (CAATCGCCGT, Sigma-Aldrich, St. Louis, MO, USA) were useful for detection of somatic hybrids and autofused plants. CAPS marker C2_At1g53670 (Tomato-EXPEN 2000, SGN 2012) linked to the *Rpi*-*mch1* gene on chromosome VII was used to verify resistant fusion plants originating from the clone *mch*/8, which was a resistant parent of mapping population (Śliwka et al. 2012). The presence of the marker C2_At1g53670 was tested in *mch*/39 parental form as well. PCR reactions for C2_At5g51970 and C2_At1g53670 were performed in a G-Storm thermocycler in 20 μl of a reaction mixture containing: 10× buffer including 20 mM MgCl_2_, 0.5 mM of each dNTP, 12.5 mM primers, 0.05 U/μl DreamTaq polymerase (Fermentas Life Sciences, Thermo Fischer Scientific Inc) and 20 ng DNA template. Amplification was performed with initial denaturation at 94 °C for 30 s followed by 40 cycles at 94 °C for 45 s, 55 °C for 45 s, 72 °C for 1 min 30 s and one final extension at 72 °C for 10 min. Digestions of the amplicons with restriction endonuclease *MspI* (Fermentas Life Sciences, Thermo Fischer Scientific Inc.) and *RsaI* (Fermentas Life Sciences, Thermo Fischer Scientific Inc.) were performed according to producers’ protocol for C2_At5g51970 and C2_At1g53670, respectively. Product sizes of selected markers are presented in Table 2.

Amplification of OPA11 was performed in a G-Storm thermocycler in 20 μl of a reaction mixture containing: 10× buffer including 20 mM MgCl_2_, 0.5 mM of each dNTP, 12.5 mM primer, 0.05 U/μl U DreamTaq polymerase (Fermentas Life Sciences, Thermo Fischer Scientific Inc) and 30 ng DNA template. The PCR parameters were: 94 °C for 30 s followed by 45 cycles at 92 °C for 15 s, 36 °C for 25 s, 72 °C for 74 s and one final extension at 72 °C for 5 min.

The amplification products of CAPS and RAPD markers were separated in 1.5 % agarose gel during electrophoresis in 1× TBE buffer (Tris-Borate-EDTA) and stained with ethidium bromide. 1 kb DNA ladder (Invitrogen) was used as a molecular marker.

### Morphological, physiological and cytological characterization

The most vigorous plants were multiplied in vitro and transferred to the greenhouse. For each formed plantlet first copy was kept in vitro and after root formation a second one was planted in pot in the greenhouse. In vitro plants with abnormalities in the growth or low vitality were eliminated. Phenotypes of vigorous greenhouse-grown plants were compared to parental forms and characterized in terms of habit, shape of leaves and whole plants, flowering and tuberization. The ploidy level mostly was evaluated by counting chloroplasts in the guard cells and for chosen group by the use of flow cytometry (Thieme et al. 1997). The mean number of chloroplasts in the pair of guard cells is assumed for diploids 11.2 (range 7.5–14.0), for triploids 14.4 (range 10.7–19.0) and for tetraploid forms 19.7 (range 16.0–25.7), respectively (Rothacker and Junges 1966). Pollen fertility was estimated using an indirect lactofuchsin method based on percentage of regularly shaped and stained pollen grains (Janssen and Hermsen 1976).

### Late blight resistance assessment

**Fig. 1:**
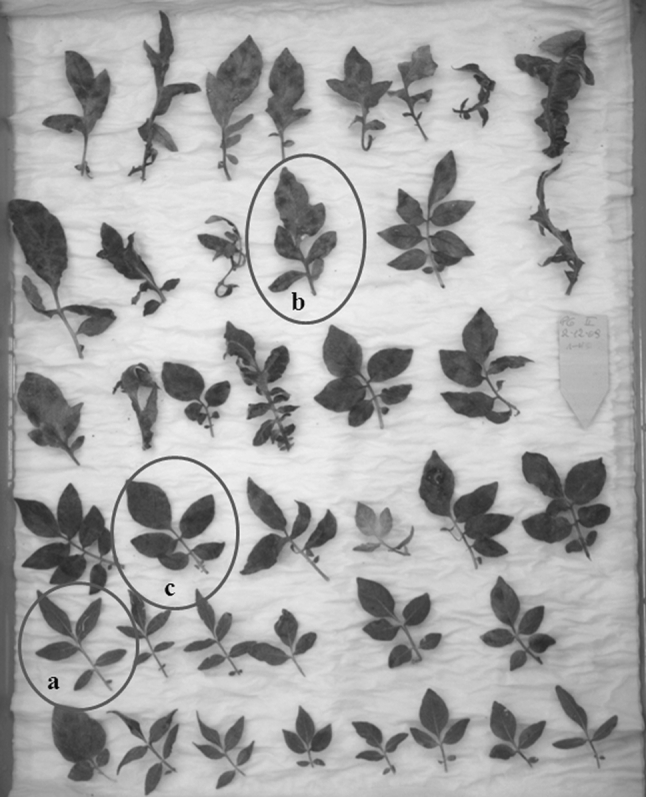
Laboratory leaf test of resistance to late blight. Leaves of somatic fusions products were inoculated and the photograph was taken 6 days later when the disease symptoms were clearly visible. *a* resistant; *b* mid-resistant; *c* susceptible example

The resistance tests of post-fusion plants, their parental forms and standard cultivars were performed in 3 years—2009, 2010 and 2011. In 2009 and 2010 resistance to *P. infestans* was tested on greenhouse plants grown from in vitro plants and in 2011 on plants grown from tubers. Greenhouse-grown plants were assessed in a laboratory test for resistance to foliage blight using detached leaf test (Fig. 1). Respective parental forms and standard cultivars were always tested together with post-fusion plants. Tests were performed at two different dates in two replicates. The single replicate was represented by one leaf in which 1–6 leaflets were evaluated based on 1–9 scale (where 9 = the most resistant). In total about 24 leaflets were tested for each genotype during season. A genotype with mean infection score ≥6 was assumed to be resistant to *P. infestans* based on research done on the *mch* by Śliwka et al. (2012). In 2009 and 2010 plants grown in the greenhouse directly from in vitro were tested with two *P. infestans* isolates MP847 and MP921. In 2011 plants were propagated from tubers harvested in previous season and tests were performed with the isolate MP921. The isolates of *P. infestans* MP847 and MP921 originated from pathogen collection of IHAR-PIB, Młochów. Those isolates were of A2 sexual type and Ia mitochondrial type, sensitive to metalaxyl, of complex race: 1. 3. 4. 7. 10. 11 (Śliwka et al. 2012). Inoculation was done by spraying (Kuhl et al. 2001) with 50 sporangia/μl suspension prepared according to Zarzycka (2001).

## Results

### Protoplast fusion

In total, 18,775 calli were produced from post-fusion products in the all eight combinations. From those calli in total 1,482 in vitro plants were regenerated (Table 1). Out of this group 344 plants were selected for further experiments.

**Table 1:** Results of development of callus, plant regeneration and production of hybrids and autofused 4*x* *mch* after protoplast fusion between *mch*/8, *mch*/39 and diploid potato lines or a cultivar

Combination	Number of					
	Calluses	Calluses with formed shoots	Somatic hybrids at ploidy level		Somatic hybrids producing tubers	Autofused 4*x* *mch*
			4*x*	>4*x*		
*mch*/8 (+) DG 81-68	2,725	113	0	0	0	24
*mch*/8 (+) DG 88-89	5,250	258	0	0	0	38
*mch*/8 (+) dH Bard	4,500	40	4	0	4	11
*mch*/8 (+) cv. Rywal	3,375	253	0	3	3	23
*mch*/39 (+) DG 81-68	850	336	106	46	50	9
*mch*/39 (+) DG 88-89	750	154	1	0	1	6
*mch*/39 (+) dH Bard	825	280	49	7	48	4
*mch*/39 (+) cv. Rywal	500	48	0	12	12	1
In total	18,775	1,482	160	68	118	116

### Identification of somatic hybrids by molecular markers and selection of 4*x* autofused *mch*

**Fig. 2:**
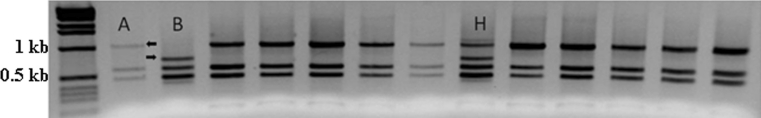
Identification of a somatic hybrid (*H*) after protoplast fusion of *mch*/8 (*A*) and a diploid line dH Bard (*B*) by the C2_At5g51970 CAPS marker digested with MspI. Remaining forms are fusion products with *mch* profile. *Arrows* indicate the marker bands

A number of obtained somatic hybrids and autofused 4*x* *mch* are presented in Table 1. In order to identify somatic hybrids and 4*x* *mch*, all putative ones from eight fusion combinations were analyzed using three SSR, two CAPS markers and one RAPD marker (Table 2). The hybrid nature of 123 plants from *mch*/39 (+) DG 81-68 fusion combination was confirmed in JKI using three SSR markers: ST13ST (122 hybrids), STI057 (123 hybrids), STM1049 (122 hybrids). Second set of 59 plants from this combination was tested in IHAR-PIB with CAPS and RAPD markers C2_At5g51970 (29) and OPA11 (29 hybrids) and next 29 hybrids were identified. In two fusion combinations of *mch*/8 (+) DG 81-68 and *mch*/8 (+) DG 88-89 somatic hybrids were not obtained. From the fusion combination of *mch*/8 (+) dH Bard, out of 25 analyzed plants four were identified as hybrids. In *mch*/8 (+) cv. Rywal, only three out of 122 analyzed plants were of expected hybrid nature. The number of somatic hybrids from next three combinations *mch*/39 (+) DG 88-89, *mch*/39 (+) dH Bard and *mch*/39 (+) cv. Rywal was 1 (out of 42 tested), 56 (out of 153 tested) and 12 (out of 31 tested), respectively (Table 3). In total 228 somatic hybrids were identified across eight combinations studied. An example of hybrid identification by a CAPS marker is demonstrated in Fig. 2. In addition, 116 autofused forms of 4*x* *mch* were obtained both from *mch*/8 and *mch*/39 parents. Seven somatic hybrids from two combinations with *mch*/8 parent were tested using C2_At1g53670 marker linked to *Rpi*-*mch1* gene (Śliwka et al. 2012). One from seven analyzed hybrids had the marker; however, all the seven were susceptible to late blight. The presence of the marker C2_At1g53670 was not found in the second parental form *mch*/39, thus this marker was not used to verify resistant fusion products originating from the clone *mch*/39.

**Table 2:** Details of markers used for identification of somatic hybrids and 4*x mch*

Marker	Marker type	Primer sequence	Product size [bp]
ST13ST	SSR	F: 5′-TTTGGGGTTCTAAATTTTAGG-3′ R: 5′-CAACCAAAATATGAATTCGTC-3′	200
STI 057	SSR	F: 5′-CCTTGTAGAACAGCAGTGGTC-3′ R: 5′-TCCGCCAAGACTGATGCA-3′	190
STM 1049	SSR	F: 5′-CTACCAGTTTGTTGATTGTGGTG-3′ R: 5′-AGGGACTTTAATTTGTTGGACG-3′	200
C2_At5g51970	CAPS	F: 5′-ACGCGAGTTTTGACTGTGCTGG-3′ R: 5′-TCTTCTTGAGAGAATCCAAACCTGTG-3′	1,000; 750
C2_At1g53670	CAPS	F: 5′-AAGGGTACAGAACGGGCATTCAC-3′ R: 5′-TGTTCCAGGGGTCTTACTGTTCCAG-3′	1,200
OPA11	RAPD	5′-CAATCGCCGT-3′	2,000; 900

**Table 3:** Identification of somatic hybrids and 4*x* *mch* by SSR, CAPS and RAPD markers (including markers for the presence of the *Rpi*-*mch1* gene)

Fusin combination of somatic hybrids and 4*x mch*	No. of tested somatic hybrids and 4*x* *mch*	Presence of markers					
		ST13ST	STI 057	STM 1049	C2_At5g51970	C2_At1g53670 for *Rpi*-*mch1* identification	OPA11
Somatic hybrids							
*mch*/8 (+) dH Bard	4				4	1	4
*mch*/8 (+) cv. Rywal	3				3	0	3
*mch*/39 (+) DG 81-68 (IHAR)	29				29		29
*mch/*39 (+) DG 81-68 (JKI)	123	122	123	122			
*mch*/39 (+) DG 88-89	1				1		1
*mch*/39 (+) dH Bard	56				56		56
*mch*/39 (+) cv. Rywal	12				12		12
4*x mch*							
*mch/8* (+)DG 81-68	24				24	24	24
*mch*/8 (+) DG 88-89	38				38	38	38
*mch*/8 (+) dH Bard	11				11	11	11
*mch*/8 (+) cv. Rywal	23				23	23	23
*mch*/39 (+) DG 81-68	9				9		9
*mch*/39 (+) DG 88-89	6				6		6
*mch*/39 (+) dH Bard	4				4		4
*mch*/39 (+) cv. Rywal	1				1		1

### Morphological, physiological and cytological characterization

**Fig. 3:**
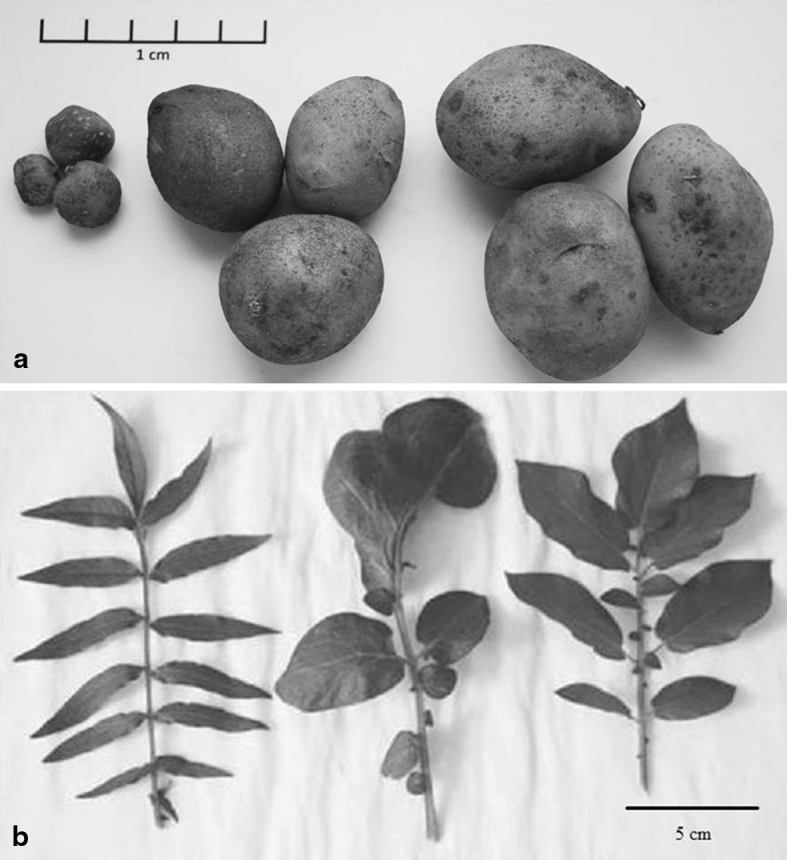
Exemplary morphology of **a** tubers and **b** leaves of somatic hybrid and its parents: *mch*/39, *mch*/39 (+) dH Bard and dH Bard (from *left* to *right*, respectively)

Morphological observations indicated two distinct groups of plants: one with intermediate phenotype, between parental forms and a second one similar to *mch* parent. Among plants with intermediate phenotype, some deformations of whole plants, leaves and tubers or various colours of flowers were observed; however, plants and tubers without these deformations were often observed (Fig. 3). Plants similar to *mch* parents were more luxuriant and had bigger leaves with widen leaflets in comparison to wild forms. Within set of 228 confirmed hybrids, 160 hybrids were tetraploids and 68 were with ploidy level higher than 4*x* (6*x* or 8*x*) (Table 1), what was confirmed by counting of chloroplasts in the guard cells or by flow cytometry. Among 118 tuberizing hybrids, 103 ones were 4*x* and the ploidy level of 15 forms were more than 4*x* (they originated from fusion of *mch* to 4*x* cv. Rywal). The separate set was represented by 116 autofused 4*x* *mch*, which was evidenced by morphological traits and molecular markers. Majority of somatic hybrids and 4*x* *mch* flowered intensively. Among them, 85 of 4*x* somatic hybrids and 113 of autofused 4*x* *mch* produced pollen grains which were successfully stained. Stainability of pollen grains of resistant somatic hybrids and 4*x* *mch* was between 40–60 and 40–70 %, respectively.

### Late blight resistance test

In 2009 and 2010 plants tested for late blight resistance were replanted in the greenhouse from in vitro. In 2011 tested plants grown from tubers. In 2009–2011 mean resistance to *P. infestans* of parental clones of *mch*/8 and *mch*/39 was scored as 8.1 and 8.0, respectively, with individual scores ranging from 4.0 to 9.0 (Table 4). The mean scores of resistance to late blight of susceptible clones DG 81-68, DG 88-89, dH Bard and cv. Rywal in 2009–2011 were 2.3, 2.1, 1.3, and 1.4, accordingly with a range 1.0–6.0 for individual readings (Table 4). In 2009–2011 in total 118 defined somatic hybrids and 116 autofused 4*x mch* were tested for resistance to late blight (Table 5). After two seasons of testing three somatic hybrids and 109 autofused 4*x mch* were defined as resistant to *P. infestans*. The mean scores of resistance to *P. infestans* of three resistant somatic hybrids were: 7.0, 7.6, and 6.9 which were slightly lower than the scores of their resistant parents. Resistance of remaining susceptible somatic hybrids ranged between 1.0 and 5.0. Mean resistance to late blight of autofused 91 4*x mch*/8 and 18 4*x mch*/39 plants was 7.5 and 7.8, respectively with a range between 5.0 and 9.0 (Table 4).

**Table 4:** Late blight resistance of parental forms, three resistant somatic hybrids and two sets of autofused 4*x* *mch* using detached leaf tests in two replicates in 2009–2011 (in scale 1–9, where 9- resistant)

Parental forms, resistant somatic hybrids and 4*x mch*	Mean resistance to *P. infestans* 2009–2011^a^	Resistance range
*mch*/8	8.1 ± 0.7	5.0–9.0
*mch*/39	8.0 ± 1.5	4.0–9.0
DG 81-68	2.3 ± 2.1	1.0–6.0
DG 88-89	2.1 ± 2.4	1.0–5.0
dH Bard	1.3 ± 0.8	1.0–3.0
cv. Rywal	1.4 ± 1.0	1.0–4.0
4*x* [*mch*/39 (+) DG 81-68]	7.0 ± 1.0	4.0–9.0
4*x* [*mch*/39 (+) dH Bard]	7.6 ± 1.5	4.0–9.0
6*x* [*mch*/39 (+) cv. Rywal]	6.9 ± 2.0	4.0–9.0
autofused 4*x mch*/8 (91 forms)	7.5 ± 0.7	5.0–9.0
Autofused 4*x mch*/39 (18 forms)	7.8 ± 0.9	5.0–9.0

^a^± Standard deviation

**Table 5:** Assessment of resistance to late blight of somatic hybrids and 4*x* *mch* using detached leaf tests for plants grown from in vitro in 2009–2010 and plants grown from tubers in 2011. Forms with resistance ≥6.0 in 1**–**9 scale were classified as resistant to *P. infestans*

Combination	No. of resistant somatic hybrids/somatic hybrids tested		No. of resistant 4*x mch*/4*x mch* tested	
	2009–2010 grown from in vitro plants	2011 grown from tubers	2009–2010 grown from in vitro plants	2011 grown from tubers
*mch*/8 (+) DG 81-68	0/0	0/0	24/24	23/23
*mch*/8 (+) DG 88-89	0/0	0/0	38/38	37/37
*mch*/8 (+) dH Bard	0/4	0/4	11/11	11/11
*mch*/8 (+) cv. Rywal	0/3	0/3	23/23	20/20
*mch*/39 (+) DG 81-68	0/46	1/46	9/9	9/9
*mch*/39 (+) DG 88-89	1/1	0/0	6/6	5/5
*mch*/39 (+) dH Bard	1/52	1/49	4/4	4/4
*mch*/39 (+) cv. Rywal	0/12	1/12	1/1	0/0
In total	2/118	3/114	116/116	109/109

## Discussion

Many wild potato species are potential sources of resistance to *P. infestans* (Hawkes 1990). At present, there is a deficit of potato cultivars resistant to late blight because new and more aggressive pathogenic races of *P. infestans* often overcome monogenic resistance (Thieme et al. 2010). Introduction of late blight resistance genes from more distantly related germplasm of wild species into the cultivated potato could be the most effective and environment friendly way to defeat *P. infestans*. Late blight resistance genes from *S. demissum*, *S. chacoense,* and *S. phureja* were successfully transferred to cultivated potato via sexual crosses (reviewed by Heřmanová et al. 2007). Majority of wild potato species are diploids (2*n* = 2*x* = 24) with 1 or 2 EBN. Unfortunately, 1EBN diploid species are not crossable to *tbr* due to different ploidy level and EBN. Hence, 1EBN forms cannot be directly used for widening the cultivated potato genepool, which is 4EBN. Because of genetic incompatibilities and the reproductive isolation between wild and cultivated potato species, genetic improvement through interspecific hybridization is difficult and time consuming. However, sexual discordances might be bypassed in several ways: through cloning of the R gene and its transfer via cisgenesis or transgenesis into potato genepool, applying protoplast fusion or changing ploidy level by doubling of chromosome number (Park et al. 2009). Many resistance Rpi genes from wild species have been identified (Park et al. 2009). About 20 Rpi genes have been cloned (Śliwka and Zimnoch-Guzowska 2012) and part of them were transferred to the cultivated potato through the cisgenic approach, like: *RB*/*Rpi*-*blb1* (Song et al. 2003; van der Vossen et al. 2003), *Rpi*-*vnt1.1* (Foster et al. 2009) and *Rpi*-*mcq1* (GMO notification B/GB/10/R29/01). Somatic hybridization is an alternative way to such R genes transfer. However, there are several bottle necks in application of somatic hybridization technique to the enrichment of breeding genepool. In somatic hybridization nuclear and cytoplasmic genomes of wild potato species are fused to potato genepool and not only the gene of interest as it is in genetic transformation. Thus, somatic fusion method is slower than genetic transformation because of necessity of elimination of unwanted traits from somatic hybrids through series of backcrosses to *tbr* background (Thieme et al. 1997; Laferriere et al. 1999). Genetic instability, lack or poor of fertility and very low crossability of produced somatic hybrids to cultivated genepool are the major problems in routine application of this technique in breeding schemes (Mattheij et al. 1992; Rokka et al. 1995; Nyman and Waara 1997; Carputo et al. 1998; Orczyk et al. 2003). Nevertheless, there are many examples of successful introgression of some agronomically important traits to somatic hybrids. Somatic hybrids resistant to *P. infestans*, *Erwinia carotovora*, *Ralstonia solanacea*, PLRV, PVX, PVY, nematode and somatic hybrids with frost tolerance and higher capacity to cold acclimation or with higher starch content and reduced concentration of glycoalkaloids are known (reviewed by Orczyk et al. 2003).

Post-fusion mixture besides products of heterofusion (heterokaryons) contains both fusion components and products of homofusion (homokaryons). Those additional products of somatic hybridization are eliminated in first steps of this process as a result of conscious selection of heterokaryons. Fusions of protoplasts of wild plants result in production of wild forms with higher ploidy level. Electrofusion and PEG induced fusion are equally effective methods (Orczyk et al. 2003) and the result of protoplast fusion mainly depends on efficient fusion protocol. Przetakiewicz et al. (2007) produced somatic hybrids between diploid interspecific hybrids using PEG method. Szczerbakowa et al. (2010) applied this approach to obtain the first set of 13 somatic hybrids between clone *mch*/8 and clone DG 81-68. In the presented study about 15 % of regenerated plants were somatic hybrids and about 8 % were autofused 4*x* *mch* via electrofusion method. Remaining 77 % plants were 2*x* *mch*, 2*x* *tbr* or 4*x* *tbr* and were eliminated. In the group of somatic hybrids characterized by intermediate phenotype, between parental forms some deformations of whole plants, leaves and tubers or various colours of flowers were observed. It could be caused by several factors: additive or non-additive effects of parental genes, new interactions between nuclear and cytoplasmic genomes or dosage of parental genome (Cardi 1998). The ploidy level was estimated by counting the chloroplasts in guard cells (Carrasco et al. 2000; Szczerbakowa et al. 2010) and by flow cytometry (Rokka et al. 1995; Thieme et al. 1997) and these two methods allowed us to identify a set of expected tetraploids, hexaploids and hybrids with higher chromosome number.

Based on published data by Śliwka et al. (2012), the presence of the *Rpi*-*mch1* gene (on chromosome VII of *mch*/8 parent) was tested using C2_At1g53670 CAPS marker linked to this gene (at a distance of 5.7 cM) in seven, assessed as susceptible to *P. infestans,* somatic hybrids originated from *mch*/8 parent. Among them one susceptible hybrid had a marker. This might have resulted from chromosome elimination/recombination or silencing of the gene (Orczyk et al. 2003). Lack of the marker in remaining six plants might have resulted from the loss of the chromosome VII or its fragment with *Rpi*-*mch1* gene in the electrofusion process, what is a frequent phenomenon in somatic hybrids’ genome (Śliwka et al. 2012). To explain this situation research has to be conducted on characterization of the chromosomal structure of obtained somatic hybrids, what is in our future plans.

It is known that nuclear–cytoplasmic interactions are important factors in male fertility (Nyman and Waara 1997; Orczyk et al. 2003). Although a number of somatic hybrids produced a lot of flowers, it was difficult to obtain a backcross generation with cultivated potato. Some of the obtained somatic hybrids have been fertile and could be backcrossed sexually to cultivated potato. However, such forms are genetically instable and very often cannot be used in further breeding exploitation. In our work the frequency of stained pollen grains of the somatic hybrids ranged between 10 and 85 %. Similar data were noted by Thieme et al. (2010). In our study flowering was abundant, 85 somatic hybrids flowered and produced stainable pollen grains and three resistant somatic hybrids had fertile pollen grains adequate for using them as pollinators. All 116 autofused 4*x mch* flowered and 113 of them produced stainable pollen grains. 4*x mch* could potentially be crossed with *tbr* plants, because one of the crossing barriers was eliminated.

Somatic hybrids produced in our study varied in morphology and resistance to *P. infestans.* This phenomenon can be partly explained by various ploidy levels of regenerants and new composition of somatic hybrids’ genome. The range of late blight resistance observed in somatic hybrids forms and 4*x* *mch* was from 1.0 to 9.0. Two classes of resistance were distinguished after resistance tests: resistant (mean scores ≥6) and susceptible forms (mean scores <6) (Table 5). The resistance level of three identified resistant somatic hybrids was enhanced, but slightly lower than resistance level of *mch* parents and similar or lower than resistance of two sets of autofused 4*x* *mch*. Only three from 118 tested somatic hybrids were resistant to *P. infestans*, while in the group of autofused 4*x mch* all forms (in total 109 after 2 years of testing) were resistant (Tables 4, 5). Resistance of assessed forms was confirmed in tests in 2012. In previous study Szczerbakowa et al. (2010) identified one resistant somatic hybrid *mch* (+) *tbr* (with mean score = 6.3) out of 10 evaluated. This resistant clone did not flower and could not be used in backcrossing programme. It is a question for our further studies why among identified somatic hybrids the frequency of those with good expression of resistance to late blight was so low.

Work is currently in progress to introgress resistance to *P. infestans* both from resistant somatic hybrids and autofused 4*x* *mch* into cultivated potato by sexual crosses. We also assume possibility of cloning of the *Rpi*-*mch1* gene for further use in cisgenic approach. In order to confirm results of laboratory test for resistance to foliage blight we plan to assess late blight resistance under field conditions. Pyramiding of R genes is common tendency in many breeding programs, since it can provide high and durable resistance to *P. infestans*. *Mch* is one of interesting and valuable source of resistance to *P. infestans*. This species has not been used in breeding so far. A resistance gene *Rpi*-*mch1* (Śliwka et al. 2012), against late blight was mapped on chromosome VII of the resistant parent *mch*/8. On potato chromosome VII only two resistance genes were mapped: *Rpi*-*mch1* (Śliwka et al. 2012) and *Rpi1* (Kuhl et al. 2001). *Rpi*-*mch1* is located on different chromosomes than other R genes (except *Rpi1*) and it can demonstrate distinct structure, mechanism of action or it can react to different effectors of pathogen. *Mch* can be valuable component in pyramiding of R genes via sexual crosses or cisgenic approach.

## Abbreviations

CAPS: Cleaved amplified polymorphic sequences
EBN: Endosperm balance number
IHAR-PIB: Plant Breeding and Acclimatization Institute-National Research Institute
JKI: Julius Kühn-Institute
PLRV: Potato leafroll virus
PSTV: Potato spindle tuber viroid
PVX: Potato virus X
PVY: Potato virus Y
RAPD: Random amplified polymorphic DNA
SSR: Simple sequence repeat
